# Emotion Regulation of Hippocampus Using Real-Time fMRI Neurofeedback in Healthy Human

**DOI:** 10.3389/fnhum.2019.00242

**Published:** 2019-07-16

**Authors:** Yashuo Zhu, Hui Gao, Li Tong, ZhongLin Li, Linyuan Wang, Chi Zhang, Qiang Yang, Bin Yan

**Affiliations:** ^1^PLA Strategy Support Force Information Engineering University, Communication Engineering College, Zhengzhou, China; ^2^Department of Radiology, Zhengzhou University People’s Hospital and Henan Provincial People’s Hospital, Zhengzhou, China

**Keywords:** hippocampus, real-time fMRI neurofeedback, emotion, regulation, autobiographical memories

## Abstract

Real-time functional magnetic resonance imaging neurofeedback (rtfMRI-NF) is a prospective tool to enhance the emotion regulation capability of participants and to alleviate their emotional disorders. The hippocampus is a key brain region in the emotional brain network and plays a significant role in social cognition and emotion processing in the brain. However, few studies have focused on the emotion NF of the hippocampus. This study investigated the feasibility of NF training of healthy participants to self-regulate the activation of the hippocampus and assessed the effect of rtfMRI-NF on the hippocampus before and after training. Twenty-six right-handed healthy volunteers were randomly assigned to the experimental group receiving hippocampal rtfMRI-NF (*n* = 13) and the control group (CG) receiving rtfMRI-NF from the intraparietal sulcus rtfMRI-NF (*n* = 13) and completed a total of four NF runs. The hippocampus and the intraparietal sulcus were defined based on the Montreal Neurological Institute (MNI) standard template, and NF signal was measured as a percent signal change relative to the baseline obtained by averaging the fMRI signal for the preceding 20 s long rest block. NF signal (percent signal change) was updated every 2 s and was displayed on the screen. The amplitude of low-frequency fluctuation and regional homogeneity values was calculated to evaluate the effects of NF on spontaneous neural activity in resting-state fMRI. A standard general linear model (GLM) analysis was separately conducted for each fMRI NF run. Results showed that the activation of hippocampus increased after four NF training runs. The hippocampal activity of the experiment group participants was higher than that of the CG. They also showed elevated hippocampal activity and the greater amygdala–hippocampus connectivity. The anterior temporal lobe, parahippocampal gyrus, hippocampus, and amygdala of brain regions associated with emotional processing were activated during training. We presented a proof-of-concept study using rtfMRI-NF for hippocampus up-regulation in the recall of positive autobiographical memories. The current study may provide a new method to regulate our emotions and can potentially be applied to the clinical treatment of emotional disorders.

## Introduction

Emotion plays a significant role in our daily lives. Our life is colorful through emotion (Dixon et al., 2017). However, the negative emotion has a bad effect on our life. The more we let our negative thoughts occupy our mind without interruption, the more interconnected they will become. Emotional disorders have become a huge threat to human mental health. It includes major depressive disorder (MMD), anxiety disorder (hypochondriasis), and other affective disorders, and patients’ symptoms usually occur during emotional fluctuation or in calm state (English et al., 2012). MDD is a common disorder which can be disabling. About two-thirds of MMD patients do not receive adequate treatment in drug therapy and psychological interventions, and only a few of them get symptom relief (Young et al., 2018). These emotional disorders may affect people’s work efficiency, causing them to live with the pain and induce disharmony with society. How we regulate our emotions matters: Our well-being is inextricably linked to our emotions. Emotion regulation is a crucial skill associated with well-being and mental health in general.

Real-time functional magnetic resonance imaging neurofeedback (rtfMRI-NF) as a potential method to improve the capability of emotion regulation and alleviate neuropsychiatric disorder symptoms has rapidly developed in recent years (Ruiz et al., 2014). Various brain regions can be used for rtfMRI-NF-augmented emotion regulation training because of their differing involvement in emotion processing and emotion regulation (Morawetz et al., 2017). The activity of some regions is associated with the intensity of emotional experiencing; the activity of other regions is associated with the implementation of emotion regulation strategies (Raffael and Reviews, 2009; Paret et al., 2011). For example, the amygdala and anterior insula (AI) have been repeatedly found to be involved in experiencing both positive and negative emotions (Sergerie et al., 2008; Kurth et al., 2010). Thus, the amygdala or AI activity levels can be taken as correlates of emotional experiencing intensity, regardless of the emotional valence, i.e., regardless of whether the person experiences positive or negative emotions. On the other hand, lateral prefrontal cortex (LPFC) regions have been repeatedly found to be involved in cognitive emotion regulation (Kohn et al., 2014). Thus, LPFC activity levels can be taken as correlates of cognitive emotion regulation efforts. NF-aided emotion regulation can be studied in healthy people, but the clinical goal of rtfMRI-NF is to promote emotion regulation in patients whose ability to regulate emotions is impaired. Emotion regulation impairment is a frequently occurring challenge for patients with different mental disorders, including mood, anxiety, stress, and personality disorders (Kring and Moran, 2008). Thus, a focus on enhancing emotion regulation is a key feature in therapy for many psychiatric patients. At the same time, the type of emotion regulation impairment can differ among patient groups (i.e., different brain regions can be affected or the same regions can be affected in a different way). For example, patients with depression show an exaggerated amygdala response to negative stimuli and an attenuated amygdala response to positive stimuli (Groenewold et al., 2013). Accordingly, the goal of rtfMRI-NF in patients with depression could be to decrease amygdala activity during the experience of negative emotions or to promote amygdala activity while experiencing positive emotions. It has been used in clinical application. For example, some research reduced the symptoms of depression by up-regulating of amygdala. Studies using this technique have demonstrated that it may be possible to successfully manipulate brain areas including the anterior cingulate cortex (Weiskopf et al., 2003; Hamilton et al., 2011), the posterior cingulate cortex (Brewer and Garrison, 2014), the anterior insular cortex (Caria et al., 2007; Andrea et al., 2010; Berman et al., 2013), posterior insular cortex (Mariela et al., 2015), amygdala (Posse et al., 2003; Vadim et al., 2011; Brewer and Garrison, 2014), primary motor and somatosensory cortex cortices (Yoo and Jolesz, 2002; Berman et al., 2012), premotor area, visual cortex (Kazuhisa et al., 2011), auditory cortex (Seung-Schik et al., 2006; Haller et al., 2013), substantia nigra/ventral tegmental area (Sulzer et al., 2013), nucleus accumbens (Greer et al., 2014), and inferior frontal gyrus (Rota et al., 2010).

The hippocampus plays an important role in the system of central nervous, especially in episodic memory and spatial navigation (Squire, 1991; Buzsaki and Moser, 2013). Considerable studies have concentrated on the application of hippocampus in emotion regulation and response to positive emotional pictures or stimuli. Meanwhile, hippocampus engagement appears to be critical for emotion processing and response to positive stimuli, including autobiographical memories (AMs) (Santangelo et al., 2018). Valerio found that low-frequency activities in the hippocampus can enhance the connectivity of interhemispheric resting-state fMRI in various cortices and hippocampus (Russell Wade et al., 2017). The hippocampus, located in the medial temporal lobe and connected with the amygdala that controls emotional memory recalling and regulation (Schumacher et al., 2018); it has increased the functional connectivity with anterior cingulate or amygdala during emotional regulation and recalling of positive memory (Guzmán-Vélez et al., 2016). Brühl et al. (2014) confirmed that the hippocampus is involved in emotion regulation (Ruiz et al., 2014). His finding also suggested that the hippocampus regulation has the potential to be applied in the clinical treatment of mental disorders, such as addiction, anxiety, and depression.

The imaging basis of fMRI is based on the blood oxygenation level dependent (BOLD) effect. In recent years, this technology has been widely used in the study of diseases such as Parkinson’s disease and depression. BOLD effect depends on the magnetic properties of deoxyhemoglobin sensitivity. Both regional homogeneity (ReHo) and amplitude of low frequency fluctuation (ALFF) are based on the BOLD signal. ReHo technology can measure the local consistency of the spontaneous low-frequency oscillation signal of the brain. It means that when a functional brain area is activated, the voxel of this brain area has more time-series change consistency. ReHo increase indicates that the local brain area consistency increases. ReHo Decrease the local brain region consistency, which indicates that the abnormality of ReHo value may indicate the consistency of neuronal activity and the abnormality of synchronization, further indicating the disorder of neuronal metabolic activity. This method can better reflect the active status of whole brain. ALFF reflects the spontaneous activity of neurons. When the spontaneous activity of neurons increases, ALFF increases. When the spontaneous activity of neurons decreases, ALFF decreases. ReHo and ALFF are two different methods of fMRI post-processing. Through these two techniques, more changes in brain function can be found.

In this research, we systematically checked the influence of NF on the activity of hippocampus based on the psychological images of positive AMs. Moreover, we also assessed the influence of NF on regulation by comparing NF and the use of pure mental strategies with pseudo-NF delivery. We used the hippocampus as a region of interest (ROI) which several studies that demonstrate that the hippocampus is an important part of limbic system. We measured the primary experimental outcome using the beta weights of NF condition (regulate) and compared to the view condition (view). We supposed that there was an upward trend of the beta weights in the regulation condition from the run 1 to the run 4 compared with the view condition and this result was better in the NF group (FG) than in the control group (CG). Moreover, the result of a control region indicated that it is not affected by NF. In addition, we investigated the changes of resting-state fMRI data using ReHo (Zang et al., 2004) maps after the rtfMRI-NF, and calculated the amplitude of low frequency (ALF) fluctuations (Zou et al., 2008).

## Materials and Methods

### Participants

Thirty-one right-handed healthy volunteers [mean ± standard deviation (SD) age, 23.2 ± 1.4 years] were recruited from China National Digital Switching System Engineering and Technological Research Center. All participants were healthy and were naive to rtfMRI-NF. We randomly assigned the volunteers to two groups, namely, the experimental group (mean age = 23.7 years, *SD* = 1.3, range 22–26, 10 male, 7 female, feedback from hippocampus: EXP) and the CG (mean age = 22.6 years, *SD* = 1.4, range 21–24, 7 male, 7 female, feedback from the intraparietal sulcus: CON) (Annette Beatrix et al., 2014). Five volunteers were excluded based on the following criteria: due to two subjects unfinished the NF runs, two subjects check realignment >1.5 mm, and one subject technical problem. The Ethics Committee of Henan Provincial People’s Hospital approved the research protocol. Volunteers written informed consent to participate in the study and we gave the financial reward to them.

### Procedure

**FIGURE 1:**
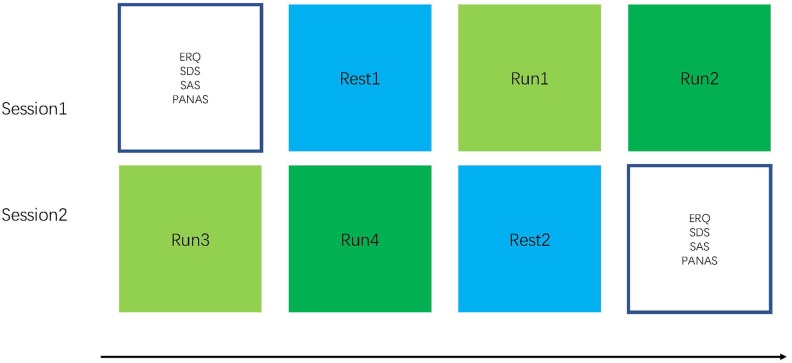
Experimental procedure: The experiment consisted of six runs with a length of 6 min 20 s each. In the beginning of the experiment, a localizer is used for hippocampus mask positioning (participants are instructed to relax). During the four training runs, the participants performed NF-based neurofeedback (NF)-based regulation of hippocampus. For the rest run, the participants are instructed to open their eyes, fixate on a cross, relax, and not think about anything in particular.

Participants were randomly assigned to the FG receiving the NF of the hippocampus (*n* = 13) and the CG provided the NF of intraparietal sulcus (*n* = 13) and completed two sessions (Young et al., 2017). To characterize emotion regulation from the baseline and impulsive traits, the participants completed questionnaires consisting of emotion regulation questionnaire (ERQ), self-rating depression scale (SDS) (Fukuda and Kobayashi, 1973), self-rating anxiety scale (SAS) (Olatunji et al., 2006), positive and negative affect scale (PANAS) (David et al., 1988), and Hamilton depression scale (HAMD). They completed one resting run and two feedback runs. The participants completed their second rtfMRI-NF session after 7 days (Figure 1). During Sessions 1 and 2, participants finished two 6 min 20 s resting-state scans in which they were asked to fixate on a cross, stay relaxed. Then, the participants completed rtfMRI-NF training after the resting-state scan (Zhonglin et al., 2016). The NF training run lasted 6 min 20 s. Participants were asked to recall positive AMs when increasing the activity of the assigned region and were instructed to keep this strategy even though they found it has no effect when increasing the ROI activity. They were suggested to concentrate on the positive AMs, the happiness in this memory, and its relation to their self-concept.

**FIGURE 2:**
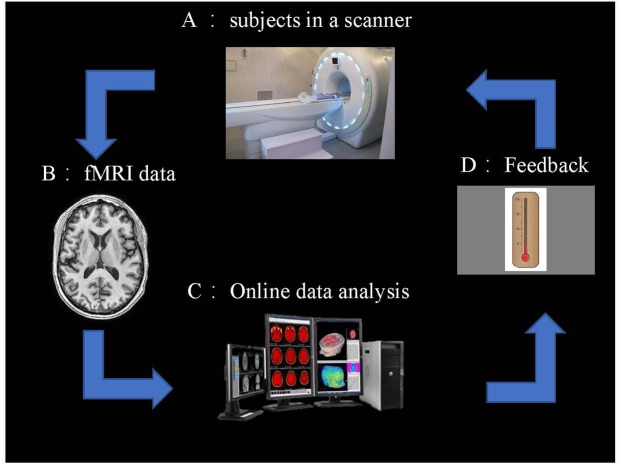
Overview of an rt-fMRI NF system. **(A)** Subjects in the fMRI scanner. **(B)** fMRI data acquisition using an echo planar imaging (EPI) pulse sequence. **(C)** Online data analysis and computation of the NF based on the BOLD responses. **(D)** NF via a scanner projection system.

The rtfMRI system: it obtains information from signals of BOLD in real-time and closed-loop. Thus, these NF signals of participants can be provided to control the activity of the brain. On the whole, the rt-fMRI system consists of: (1) the subjects, (2) fMRI data scans, (3) preprocessing of fMRI data, (4) analysis, and (5) NF (Figure 2).

### Experimental Paradigm

A practice run was applied to the participants for approximately 20 min at 1 day before scanning to familiarize them with the experimental procedure. The instructions were given to them, including the purpose of this research and the paradigm of NF. Before scanning, every participant noted down for happy AMs. In this experiment, several examples were provided to them, such as traveling, getting a good grade, and falling in love with a girl or boy. The subjects were instructed to induce positive emotions using positive memories during scanning.

**FIGURE 3:**
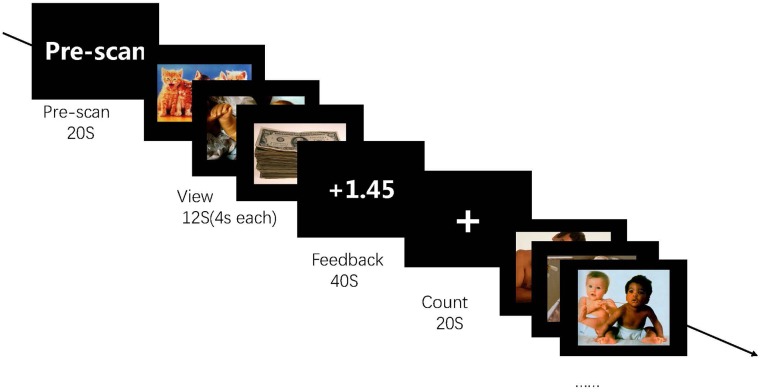
Experimental procedure in the run. The experiment consists of four runs each lasting for 6 min 20 s each, and each run contains five blocks. During the feedback run, participants experience rt-fMRI NF training consisting of alternating blocks of view, feedback, and count (instructed to count backward from 300 by a given integer). During the “feedback” blocks, they are asked to apply a recall strategy with positive AMs to regulate the ROI activity. The feedback value is computed based on ROI activation.

Participants completed four feedback runs. Every run started with a pre-scanning of 20 s to obtain magnetization equilibrium. Then, participants performed five blocks, which included view, count, and feedback conditions. For the view condition, images of positive sets were centrally presented at 4 s each, and participants tried their best to imagine experiencing the depicted situation. For the feedback condition, participants attempted to recall positive AMs that could help them up-regulate their activity of the brain region. For the count condition, participants were asked to fixate at a displayed cross and think of nothing in particular (Figure 3).

Each NF run consisted of alternating blocks of pictures, regulation, and count. Each session included two NF runs (6 min 20 s), and a rest run without NF information was provided after two training runs. The stimuli consisted of positive pictures obtained from the International Affective Picture Set. Three pictures were showed in each block lasted for 4 s (total block time is 12 s). Eighty images (mean and *SD* for normative valence 6.67 ± 0.24) were used for two positive runs. All images were displayed in Psychopy^1^.

### Online Data Analysis

**FIGURE 4:**
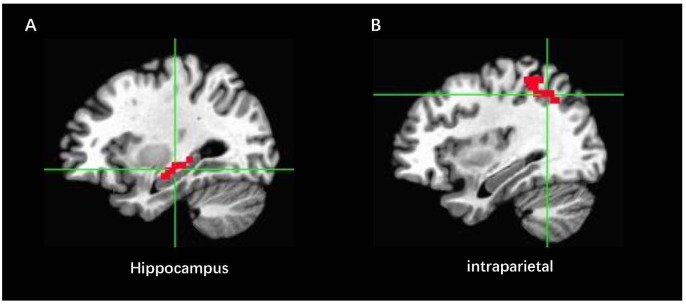
Locations for the feedback and control localizer brain regions in Talairach space: **(A)** ROI_EXP_ indicates the NF region in the right hippocampus, and **(B)** ROI_con_ represents the control NF region in the intraparietal area.

Online fMRI data were performed in Analysis of Functional NeuroImages (AFNI^2^). Before the NF procedure, a short EPI (20 s) scan and a T1image were acquired for each participant. The T1 image was transformed in Talairach space. We defined the bilateral ROI in the Talairach space. They were first turned into the original T1 space, and the short EPI scan was used to define the EPI space. Thus, the ROI mask in the EPI space was constructed (Figure 4). All volumes of EPI were volume-registered to the same single EPI volume during the rtfMRI-NF. In this way, the masks of ROI were used to all fMRI data in real time (Young et al., 2014). The volume location of all acquired volumes exported to the mean values of BOLD activity for the ROI performed by an AFNI real-time module during the rtfMRI-NF. Real-time data analysis comprised incremental 3D motion detection and correction and drift removal and resulted in incrementally computed statistical maps based on the general linear model (GLM) and event-related averages. Then, the mean signals were used to calculate the NF score and it was updated every 2 s. The NF signal of each feedback condition was computed as follows:

$${\text{Score}}_{i}=\frac{\left({\text{BOLD}}_{i}-{\text{BOLD}}_{\text{baseline}}\right)}{{\text{BOLD}}_{\text{baseline}}}\times 100,$$

where BOLD_baseline_ represents the BOLD signal of the hippocampus in the last count condition before the *i* repetition time (TR), and BOLD*_i_* represents the BOLD signal of the hippocampus in the *i* TR (*i* = 1, 2, …), which are computed every 2 s and expressed in a number (Vadim et al., 2011; Young et al., 2016).

### Data Acquisition

The fMRI data were acquired at the Imaging Center of Henan Provincial People’s Hospital. Brain imaging was performed by using a 3.0T GE Discovery MR750 scanner (General Electric, Fairfield, CT, United States) with a standard eight-channel head coil was adopted. We used sponge mats to restrict the motion of the head and diminish the noise of the scanner. fMRI data were acquired by using a standard GRE-T2^∗^ EPI sequence and the following parameters were used: field of view = 220 × 220 mm, TR = 2000 ms, TE = 30 ms, matrix size = 64 × 64, slices/slice thickness = 33/3.5 mm, and flip angle = 80°. We used a 3D fast spoiled gradient sequence for acquiring the T1-weighted image and the parameters were used: TR/TE = 8.268 ms/3.22 ms, FOV = 256 × 256 mm, matrix size = 256 × 256, slices = 156 mm, and flip angle = 12°.

### Preprocessing of fMRI Data

The preprocessing of fMRI data was performed in DPARSF^3^, which is based on SPM12 and REST^3^. Analysis of NF training fMRI data was performed through statistical parametric mapping (SPM12 ^4^). The first 10 initial scans of every fMRI runs were discarded because the unstable magnetic field can cause distortion of data. The following procedure was included in the preprocessing of data. The acquired fMRI data were corrected by slice-timing and realignment. fMRI data were transformed into the standard Montreal Neurological Institute (MNI) space by linearly registering to the anatomical data and a kernel of 6 mm FWHM was used to smooth. Finally, white matter mask, cerebrospinal fluid mask, six head motion parameters, and global mean signal were regressed out of the time series (Brown et al., 2014; Shao et al., 2014; Yao et al., 2016).

### Data Analysis

Separate models were established in SPM for the four feedback runs, and two regressors consisted of the GLMs. Three experimental regressors were “view,” “NF,” and “baseline,” and the movement vectors of the realignment were involved in the GLM model. We modeled the conditions as the blocks of brain activation by convolving with function of hemodynamic response. Then, the fMRI data were filtered by high pass (128 s).

Region of interest analysis of fMRI data was performed in SPM12. A one-sample *t*-test of the contrasts View > Baseline, Regulation > Baseline, and View > Regulation was conducted on the single level to check the effect of NF. Then, we extracted the mean contrast value from all voxels of the hippocampus and the region of control. Beta weights were extracted from all voxels of ROI. We used a paired two-sample *t*-test for the contrast Regulation > Baseline to verify our directed hypothesis that the activity of hippocampus was increased in the regulate condition when compared to the view condition. We performed the ROI analysis in the hippocampus between the FG and CG.

### Resting-State Data Analysis

#### Low Frequency Fluctuation Analysis

The DPABI was used to calculate ALFFs. To calculate ALFFs of the resting-state data, the fMRI data were smoothed using a Gaussian kernel of 8 mm (FWHM). Next, the time series was transformed into the frequency domain by a fast Fourier transform. Then we calculated the square root at each frequency in the power spectrum. ALFF values of the resting-state data were calculated in every voxel after at the frequency of the power spectrum across 0.01–0.08 Hz. We divided the ALFF of each voxel by the mean ALFF value for standardization. The *T*-test was performed in REST software (Zou et al., 2008).

#### Calculation of ReHo

Zang et al. (2004) described the specific calculation method, and the analysis of ReHo maps was performed by DPABI (Chao-Gan and Yu-Feng, 2010). ReHo computation is represented using Kendall coefficient of concordance (KCC). It may be used to explore the neuro mechanism in the resting-state. The KCC was used to calculate the ReHo maps of the time-series of a fixed voxel with the nearest 26 adjacent voxels. We divided the ReHo maps by global average value of ReHo. Then, we smoothed the values of ReHo using a kernel of 4-mm FWHM.

The ReHo maps of FG and CG were calculated with a two-sample *t*-test. We computed the maps of ReHo from the resting-state which was scanned before and after the NF training. A paired *t*-test was performed on the ReHo maps of the FG and CG, and the ReHo maps were computed from the resting-state data that collected before and after the rtfMRI-NF training.

## Results

### Behavioral Data

We compared the questionnaires before and after the training through a paired *t*-tests to analyze the effects of NF training on behavior. And the results of *t*-tests did not show remarkable change during NF training. However, the scale score of the experimental group showed a downward trend in the SAS before the training (*M* = 29.2 ± 49) compared with after the training (*M* = 2.56 ± 1.8) of the feedback group (FG). Moreover, subjects were asked to write down the methods they used for the success of regulation. These results are shown in Table 1

**Table 1:** Questionnaire score characteristics (mean ± SD).

Measures	Mean/*SD*				Group comparison	
	FG		CG		Statistics	
	Before	After	Before	After	Feedback	Control
*N*	13		13			
Age	23.7/1.3		22.5/1.4		*t* = −1.4 *p* = 0.18	
Gender	8 m/6 f		6 m/8 f			
SAS	29.2/4.9	25.6/1.8	26/6.02	22.8/2.65	Ns *p* = 0.06	Ns *p* = 0.24
SDS	27.8/4.4	26.3/1.4	23.8/3.1	22.1/1.5	Ns *p* = 0.17	Ns *p* = 0.07
ERQ_rea	32.4/3.4	34/4.5	28.3/4.2	28.9/3.7	Ns *p* = 0.26	Ns *p* = 0.16
ERQ_sup	16.3/2.5	17/4.1	15.3/3.9	16.2/4.1	Ns *p* = 0.44	Ns *p* = 0.89
PANAS(P)	27/6.1	33.1/7.9	26.1/8.6	27.4/9.6	Ns *p* = 0.34	Ns *p* = 0.14
PANAS(N)	13.7/2.8	12.3/2.7	12.4/2.1	13.2/2.6	Ns *p* = 0.22	Ns *p* = 0.16
HAMD	12.7/3.7	8.6/3.1	5.7/4.5	5.1/3.4	Ns *p* = 0.47	Ns *p* = 0.29

*Given are the mean/SD of the assessed demographic data. N, number of subjects; m, male; f, female; SAS, self-rating anxiety scale; SDS, self-rating depression scale; ERQ, emotion regulation questionnaire; rea, reappraisal sub-score; sup, suppression sub-score; PANAS, positive and negative affect scale; P, positive; N, negative; HAMD, Hamilton depression scale; Ns, not significant; t-test, two-tailed. The p-values were tested for the before- vs. after-NF testing.*

### ROI Analysis

Region of interest analysis was used to verify that the change of hippocampus activity is different from the experimental group to the CG. Two ROIs were defined as spheres of 8 mm radius in the MNI, respectively, in the left hippocampus (−25, −7, −22, *r* = 8mm) and left intraparietal sulcus (−44, −46, 47, *r* = 8mm), which were used to assess changes in BOLD activity. The beta weights of conditions (“viewing” and “regulation”) from the ROIs of FG and CG were computed by a random-effect GLM. For each condition, we used the beta weights to characterize the magnitude of ROI activation during training.

**FIGURE 5:**
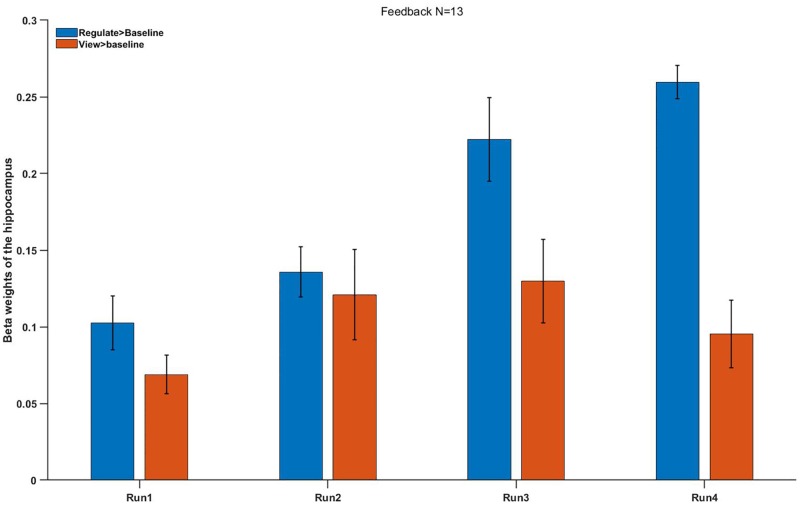
Mean beta weights of the subjects’ individual localizer hippocampus ROIs. Conditions regulate > baseline (blue) and view > baseline (red) are presented during the four runs. Error bars represent the standard errors.

The preliminary research solved the influence of repeated NF runs on hippocampus regulating in the FG (Figure 5): Left hippocampus and right hippocampus responses were remarkably higher in the FG of contrast regulate > baseline (*M* = 0.25 ± 0.21) compared with contrast view > baseline [*M* = 0.09 ± 0.14, *t*(13) = 2.65, *p* < 0.033]. In the FG, hippocampus activation had a rising trend level across the four NF runs. A *post hoc* analysis was used to directly compared the activation of hippocampus between runs 4 and 1 in the regulate conditions. The activation of the hippocampus during regulation (against baseline condition) was remarkably higher in run 4 compared with run 1: *t*(13) = 4.2, *p* = 0.013. No difference was observed between runs 1 and 4 during view > baseline (*p* = 0.814).

**FIGURE 6:**
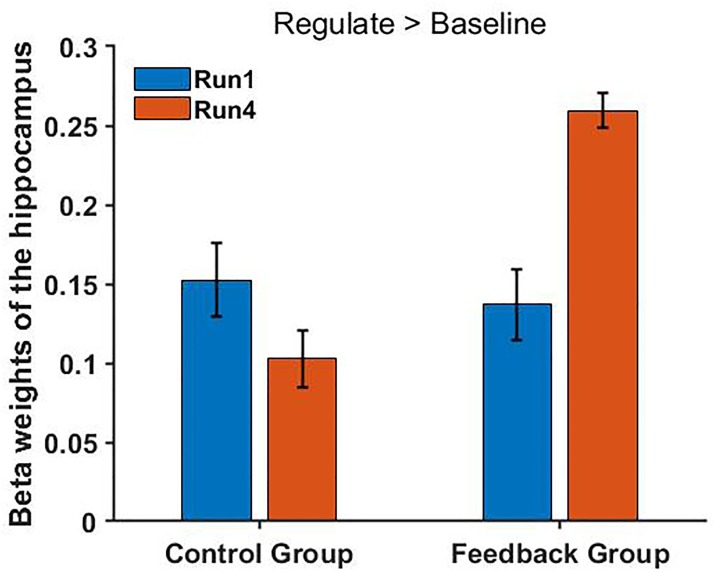
FG shows a significantly increased hippocampus activity in the main contrast (regulate > baseline) [*t*(13) = 2.756, *p* = 0.034] in the fourth run but not in the first run (regulate > view: *p* = 0.648) compared with the CG. Bars indicate the standard errors.

No significant difference was found between the FG and CG in the run 1 (regulation condition, *p* = 0.895), but we found a different trend level in the run 4 (*p* = 0.055). In conclusion, these results showed a remarkably improved hippocampus up-regulation in the FG but not in the CG (Figure 6).

In the CG, there was no remarkable differences in the contrast regulate > view, the contrast view > baseline or regulate > baseline between runs (regulate > view condition: *p* = 0.741, view condition: *p* = 0.693, regulate condition: *p* = 0.562).

The BOLD activation levels for the left intraparietal sulcus ROI are close to zero (after group averaging) and exhibit no obvious trend across runs. In the FG, there was no remarkable differences in the contrast view > baseline or regulate > baseline between runs.

### Results of ReHo

We used a two-sample *t*-test to investigate the effects in FG and CG. Statistical analysis maps were shown in Figure 6 and Table 2. The score of ReHo maps increased after the training were found in the left anterior cingulum gyrus, left superior parietal gyrus, left inferior parietal gyrus, right frontal gyrus, right angular gyrus, right precuneus, and superior frontal gyrus. The score of ReHo maps decreased after the training were found in the left parahippocampal gyrus, left hippocampus, middle temporal gyrus, right caudate nucleus, right fusiform gyrus, insula, and right postcentral gyrus.

**Table 2:** Brain regions where the ReHo map differed between the feedback group (FG) and control group (CG).

Brain region	Voxels	MNI coordinates			*t*-value
		*X*	*Y*	*Z*	
R Superior frontal gyrus	31	−15	57	27	5.55
L Middle temporal gyrus	20	−54	−21	−24	−4.39
L Anterior cingulate gyrus	24	0	−48	42	3.60
B Lingual gyrus	16	−9	−99	−21	−5.22
L Inferior occipital gyrus	34	−39	−84	3	−6.97
L Frontal lobe	35	6	54	18	4.52
R Superior temporal gyrus	56	60	−54	21	6.06
L Middle occipital gyrus	69	45	−84	−12	−4.92
R Precentral gyrus	46	54	9	33	5.82
R Middle frontal gyrus	106	45	9	57	5.55

*L, left; R, right; B, bilateral; AlphaSim corrected p < 0.01.*

**FIGURE 7:**
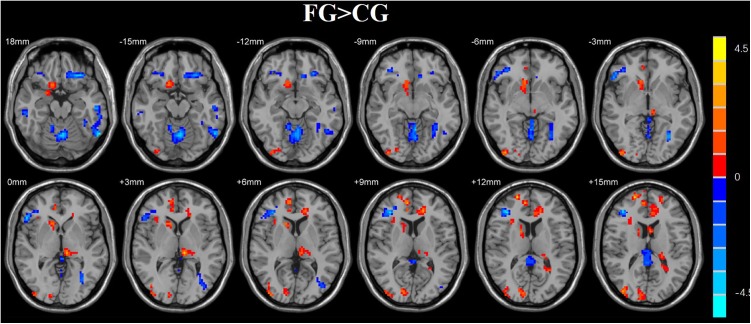
Regions with altered ReHo score in the experimental group after rtfMRI-NF training. The score of ReHo maps increased after the training were found in the left anterior cingulum gyrus, left superior parietal gyrus, left inferior parietal gyrus, right frontal gyrus, right angular gyrus, right precuneus, and superior frontal gyrus. The score of ReHo maps decreased after the training were found in the left parahippocampal gyrus, left hippocampus, middle temporal gyrus, right caudate nucleus, right fusiform gyrus, insula, and right postcentral gyrus (AlphaSim corrected *p* < 0.01).

A paired *t*-test was used in the FG. Statistical analysis maps were presented in Table 3 and Figure 7. The ReHo score of increased in the FG was observed in the right hippocampus, right parahippocampal gyrus, left insula, right superior frontal gyrus, left anterior cingulate gyrus, left inferior occipital gyrus, left inferior frontal, and right middle frontal gyrus. The regions of decreased ReHo score were found in the right cingulum, left cingulum, and left middle occipital gyrus.

**Table 3:** Brain regions of the FG where the ReHo map differed between the Rest1 and Rest2.

Brain region	Voxels	MNI coordinates			*t*-value
		*X*	*Y*	*Z*	
R Superior frontal gyrus	12	18	−15	75	−4.358
L Middle temporal gyrus	15	−51	72	24	−5.5967
B Cingulate gyrus	24	0	−48	42	−3.5991
B Frontal lobe	34	−15	57	27	5.5533
L Inferior frontal	15	−30	21	−15	4.7447
L Para hippocampal gyrus	17	27	−18	−18	4.4082
L Lingual gyrus	19	−9	−99	−21	−5.2165
R Fusiform gyrus	21	39	−48	−18	−4.5105
R Inferior occipital gyrus	34	−39	−84	−3	−6.917

*L, left; R, right; B, bilateral; AlphaSim corrected p < 0.01.*

### Results of ALFF

**FIGURE 8:**
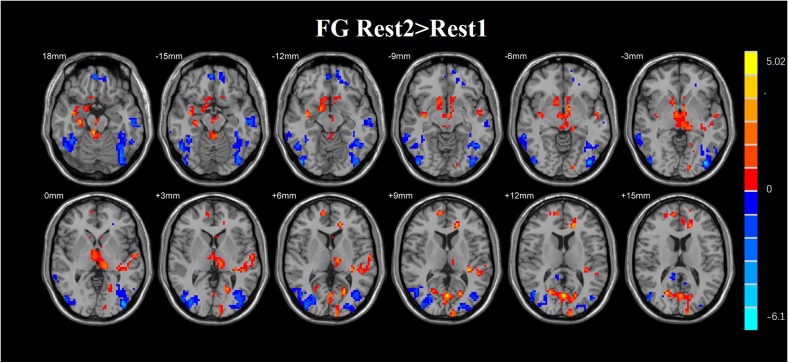
Regions with altered ReHo score in the experimental group after rtfMRI-NF training. The ReHo score of increased in the FG was observed in the right hippocampus, right parahippocampal gyrus, left insula, right superior frontal gyrus, left anterior cingulate gyrus, left inferior occipital gyrus, left inferior frontal, and right middle frontal gyrus. The regions of decreased ReHo score were found in the right cingulum, left cingulum, and left middle occipital gyrus (AlphaSim corrected *p* < 0.01).

A two-sample *t*-test was used to investigate the effects in FG and CG. The differences of the group were presented in Table 4 and Figure 8. The FG remarkably exhibited reduced ALFF in the left middle occipital gyrus, left parahippocampal gyrus, and right middle frontal gyrus compared with CG. The regions of remarkably increased ALFF was observed in the left anterior cingulate and left superior frontal gyrus.

**Table 4:** Brain regions where the ALFF differed between the FG and CG.

Brain region	Voxels	MNI coordinates			*t*-value
		*X*	*Y*	*Z*	
B Inferior frontal gyrus	57	28	21	−15	5.2198
R Hippocampus	56	20	−5	−12	4.8907
B Inferior frontal	102	26	21	−19	4.7205
R Insula	83	27	25	−11	4.6870
L Anterior cingulate	71	0	36	29	4.214
L Paracentral	759	0	−27	60	−4.5413
R Amygdala	82	21	−5	−12	4.8632
R Para hippocampal gyrus	89	27	−20	−22	3.2294

*L, left; R, right; B, bilateral; AlphaSim corrected p < 0.01.*

## Discussion

The capability to improve emotional regulation is very important to people. rtfMRI-NF training, in which BOLD fMRI data processing is concomitantly performed with data acquiring, has enabled rtfMRI-NF training in which individuals recognize and regulate the BOLD signals from their brain. Increasing evidence suggests that rtfMRI-NF has clinical utility in reducing symptoms associated with chronic pain (Zhang et al., 2009), smoking cessation (Hartwell et al., 2016), anxiety (Zilverstand et al., 2015), post-traumatic stress disorder (PTSD) (Nicholson et al., 2016), and MDD (Linden et al., 2012). The amygdala and the hippocampus are, respectively, associated with emotional processing and declarative memory (Guzmán-Vélez et al., 2016). A mature neurobiological model of emotion regulation is associated with cognitive control of emotions to prefrontal cortex areas including the amygdala and hippocampus. Many rtfMRI studies have targeted the brain region critically involved in both emotional processing and the pathophysiology of MDD, i.e., the amygdala (Young et al., 2014). The amygdala is an important part of emotion generation (Phelps and Ledoux, 2005) and regulation, and hippocampus constitutes a core structure of emotion processing and working memory through the functional coupling with the amygdala. The hippocampus is also related to NF of emotion regulation (Schumacher et al., 2018).

The goals of this study are assessing the effect of NF of hippocampal activity during four NF runs by recalling of AMs. Recall of AMs is an effective method of emotion regulation. In our study, we systematically analyzed the change of self-regulation efficacy induced by NF of ROI activity in the hippocampus based on positive AMs and sense. We also verified the feasibility of training healthy participants to improve their hippocampus BOLD activity using contemplating positive AMs during four NF runs. The FG showed remarkably higher activation of the hippocampus in the run 4 compared with the run 1 during the up-regulation of their NF. This effect of FG was better than in the CG, which is also trained with NF from the hippocampus. We also extracted the BOLD signal of the control region (intraparietal sulcus). There was no significant difference of the control region during the four NF training sessions. In the intraparietal sulcus, we did not observe NF alter the activity of the brain, which supports the hypothesis NF specificity on the hippocampus. This also demonstrated the specificity of the effect of hippocampus-based NF training. Our results indicated that individuals could voluntarily control BOLD responses in the hippocampus during emotional memory recall by using short rtfMRI-NF training.

To characterize emotion regulation from the baseline and impulsive traits, the participants completed questionnaires consisting of ERQ, SDS, SAS, PANAS, and HAMD. We compared the questionnaires before and after the training through a paired *t*-tests to analyze the effects of NF training on behavior. And the results of *t*-tests did not show remarkable change during NF training. Those results may indicate that we reduced the negative emotions of the subjects and increased the positive emotions of the subjects after NF training. No behavioral change observed. It may be due to the reason that our experiment is aimed at healthy people, and the insufficient number of NF training.

**FIGURE 9:**
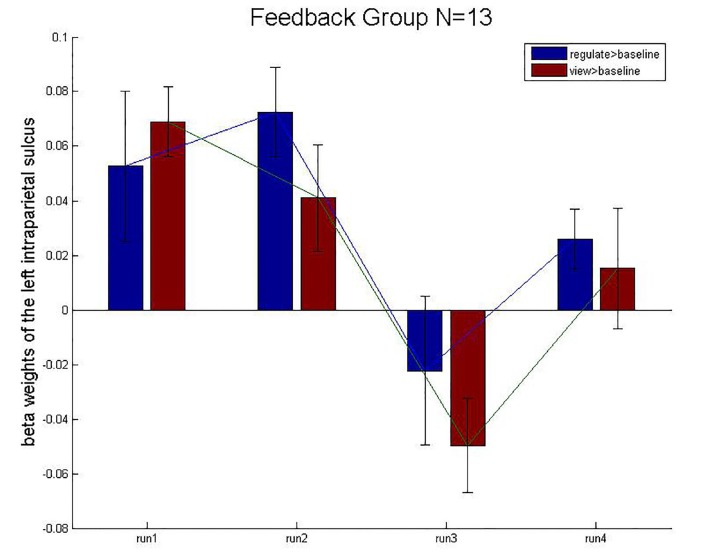
Mean beta weights of the subject’s localizer the intraparietal sulcus ROIs. Conditions regulate > baseline (blue) and view > baseline (red) are presented during the four runs. Error bars represent the standard errors.

We employed a control condition in which subjects received rtfMRI-NF from the intraparietal sulcus, a region primarily implicated in number processing. The BOLD activation levels for the left intraparietal sulcus ROI are close to zero (after group averaging) and exhibit no obvious trend across runs (Figure 9). This result suggests that the experimental and CGs differed in their NF training effects based on the specific target brain region (left hippocampus and left intraparietal sulcus). The enhanced control over left hippocampus BOLD activity appeared to specifically result from rtfMRI-induced learning. The CG underwent the same rtfMRI NF procedure as the experimental group, but received sham NF information corresponding to BOLD activity in the left intraparietal sulcus, a region that has been consistently implicated in numeric processing. Although the CG initially showed a similar level of BOLD activity in the target ROIs as the experimental group, the CG did not differentially modulate activity in either the intraparietal sulcus or the hippocampus across runs. Therefore, the observed learning effect appeared attributable to ROI-specific NF training rather than to nonspecific aspects of task performance such as repetition or practice effects.

**FIGURE 10:**
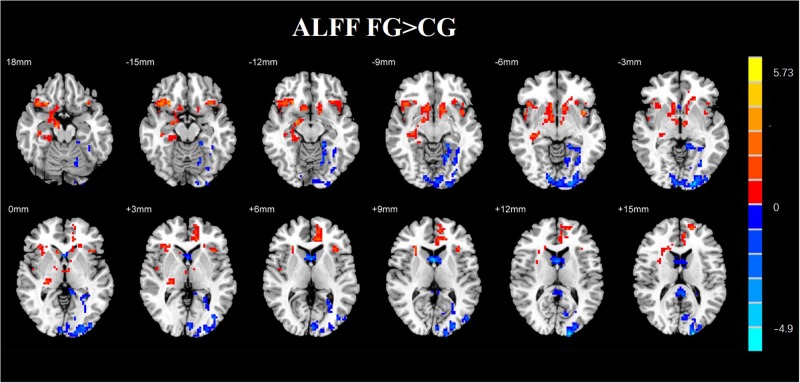
Regions with altered ALFF score comparison of the experimental and CGs. The FG remarkably exhibited reduced ALFF in the left middle occipital gyrus, left parahippocampal gyrus, and right middle frontal gyrus compared with CG. The regions of remarkably increased ALFF were observed in the left anterior cingulate and left superior frontal gyrus (AlphaSim corrected *p* < 0.01).

The imaging basis of fMRI is based on the BOLD effect. In recent years, this technology has been widely used in the study of diseases such as Parkinson’s disease and depression. BOLD effect depends on the magnetic properties of deoxyhemoglobin sensitivity. Both ReHo and ALFF are based on the BOLD signal. ReHo can measure the local consistency of the spontaneous low-frequency oscillation signal of the brain. It means that when a functional brain area is activated, the voxel of this area has more time-series change consistency. The increasing of ReHo indicates that the consistency of brain area increases. The decreasing of ReHo shows the opposite result, which means that the abnormality of ReHo value may represent the consistency of neuronal activity and the abnormality of synchronization, further indicating the disorder of neuronal metabolic activity. This method can better reflect the whole brain active status. ALFF also reflects the spontaneous activity of neurons. When the spontaneous activity of neurons increases, ALFF increases. When the spontaneous activity of neurons decreases, ALFF decreases. ReHo and ALFF are two different methods of fMRI post-processing. Through these two techniques, more changes in brain function can be found. In this study, the brain region of the ReHo value changes coincides with the brain region where the ALFF value changes. Some scholars believe that the increase in ReHo value may promote local neuronal activity. The enhancement of ReHo and ALFF values in some brain regions is also increased, which may indicate the local consistency of neuronal electrical activity in these brain regions.

### ReHo

In clinical research, the ReHo is mainly used as a brain imaging sign to explore the neural mechanism of mental illness by comparing the difference in ReHo values between mental patients and normal people, and as a means to predict and diagnose mental illness. At present, many scholars have applied the ReHo method to the study of brain diseases such as depression, schizophrenia, epilepsy, traumatic stress disorder, and anxiety. The ReHo analysis has been widely used in the study of mental disorders such as depression, traumatic stress disorder, and anxiety disorders. Wei et al. (2015) found abnormalities in the neuro activity of the hippocampus, anterior cingulate gyrus, and central posterior gyrus in depression patients. Li et al. (2017) conducted a meta-analysis of the literature on depression ReHo from 2003 to 2016. It was found that there were abnormalities of ReHo maps in the precuneus, cingulate gyrus, fusiform gyrus, postcentral gyrus, frontal lobe, and other brain regions of patients with depression.

We assessed the effect of emotion regulation using NF. Moreover, we considered the changes in ReHo maps of healthy subjects when they keep the resting-state after the NF training. The results of the ReHo indicated that there are many regions which have a statistically remarkable difference in the FG and CG. We found that the left precuneus, middle cingulum gyrus, middle frontal gyrus, insula, left inferior parietal, and superior parietal gyrus have a remarkable difference between the FG and CG. Prefrontal lobe, temporal lobe, and parahippocampal gyrus are related to emotion regulation. Compared with CG, we also detected that there was a statistically remarkable difference in the regions of ReHo map in the FG. The results indicated that the brain regions related to emotion may be regulated by the presented rtfMRI-NF.

Regional homogeneity analysis was used to investigate the effects of neuropsychiatric disorders in previous, such as MMD, BPD, and PTSD. In the research about MMD, there was a remarkable difference between healthy and depressed subjects in the fusiform gyrus, temporal lobe, amygdala, parahippocampal gyrus, and hippocampus. The regions of the brain, which were influenced by NF, were the same as the regions with unusual ReHo maps in the disorders. Our study demonstrates that rtfMRI-NF may be helpful in human beings to regulate the activity of their brain, and the analysis of ReHo provides a new method which is helpful to study the mechanism of emotion regulation.

### ALFF

We applied the resting-state data to investigate the influence of NF. On the basis of a previous research, Zou et al. (2008) defined a standard measure of ALFF. Subsequently, ALFF is used to represent different physiological states of the brain and is a helpful method to investigate the brain activities of healthy subjects. In the present study, we found that many regions of FG have a statistically remarkable difference in ALFF compared with CG. The left inferior parietal, left cingulum gyrus, middle frontal gyrus, and superior parietal gyrus showed a remarkable difference in the FG and CG.

By analyzing the experimental results, we found that most of the brain regions where the ALFF value changes are the right brain regions. In recent years, the default network has received a lot of attention. The activity of default network is enhanced in the resting state, and it is weakened in the task state. It is a brain network system with high activity in the resting state of the brain. It contains bilateral hippocampus, medial prefrontal lobe, posterior cingulate gyrus/the precuneus, and the bilateral angulation. The default network is closely related to advanced functions such as self-introspection, memory, and emotional/maintenance awareness. In this study, the brain regions with some functional changes are part of the default network. It may be due to NF training. In addition, the medial frontal cortex participates in the cognitive execution network. In this experiment, the ALFF value of the left frontal gyrus was higher than that of the CG, which the cognitive performance of the subject may be improved due to NF training (Figure 10).

The precuneus is associated with high-level cognitive functions such as contextual memory and consciousness. In the resting state, the precuneus is highly active. A study of Cabeza et al. (2003) showed that the precuneus was involved in the conscious short-term memory recall of the subjects. In our study, the ALFF value of the precuneus increased, indicating that the NF training through the hippocampus may affect the function of the emotional memory.

The cingulum gyrus brings back the output from the hippocampus, the amygdala, the orbit frontal cortex, and the medial frontal gyrus, and the nerve impulse is transmitted to the anterior cingulate and striatum, which has always been an important part of the emotional circuit. It also participates in emotional and self-evaluation processes, which are closely related to depressive symptoms. The cause of this phenomenon may be due to NF from the hippocampus, and the participants used positive AM recalls. The middle frontal gyrus participates in the cognitive execution network. In this experiment, the ALFF value of the left frontal gyrus was higher than that of the CG, which may improve the cognitive performance of the subject due to NF training. The parietal cortex plays an important role in the process of concentration. It consists mainly of the cortex that senses and monitors the response of various parts of the body to external stimuli. NF training requires the subjects to maintain a high degree of attention, monitoring the feedback information and using strategies to regulate.

The thalamus, amygdala, and hippocampus are considered to be the key brain regions of the limbic system and are closely related to the emotional memory function. The results showed that the ALFF values of the amygdala and hippocampus were increased in the experimental group compared with the CG. This may be due to that the NF training changed the emotional network of the subjects, and the local consistency was enhanced, indicating the emotional memory functions of the subjects had been improved.

To our knowledge, our findings may provide guidance for the application of NF in emotional disorders. It may help people understand the underlying working mechanism of the hippocampus.

## Conclusion

Our study demonstrated that NF training could up-regulate the activity of the hippocampus. The activation of hippocampus increases after four NF runs and alters the ReHo and ALFF of the resting-state hippocampus. The hippocampal activity of the experimental participants was higher in the FG than in the CG. Emotion regulation training by using the recall of positive AMs, which supports the hippocampus NF, is more efficient than training with the NF of intraparietal sulcus. This research is a beginning for further investigating the application of NF of the hippocampus as a promising tool in emotion regulation of healthy human. In addition, up-regulation of the hippocampus could be useful for diseases which has the problems in emotion regulation and elevated hippocampus activity. NF could be used to train the capability of emotion regulation. It can provide a potential tool to increase efficacy under these circumstances.

## Ethics Statement

This study was carried out in accordance with the recommendations of “The Ethics Committee of Henan Provincial People’s Hospital” with written informed consent from all subjects. All subjects gave written informed consent in accordance with the Declaration of Helsinki. The protocol was approved by the “The Ethics Committee of Henan Provincial People’s Hospital.”

## Author Contributions

YZ, HG, and LT conceived and designed the experiments. YZ, QY, and ZL performed the experiments. YZ and CZ analyzed the data. LW and BY contributed to reagents, materials, and analysis tools.

## Conflict of Interest Statement

The authors declare that the research was conducted in the absence of any commercial or financial relationships that could be construed as a potential conflict of interest.

## Footnotes

1 http://www.psychopy.org/

2 http://afni.nimh.nih.gov/afni/

3 http://www.restfmri.net/

4 www.fil.ion.ucl.ac.uk/spm
